# Supplementary material for: Prioritization of the Target Population for Coronavirus Disease 2019 (COVID-19) Vaccination Program in Thailand
Source: Int J Environ Res Public Health. 2021 Oct 14;18(20):10803. doi: 10.3390/ijerph182010803 (PMC8535856; doi:10.3390/ijerph182010803)
Supplement: Supplementary file 1 [file ijerph-18-10803-s001.zip › ijerph-1367387-supplementary.pdf]

Note

1. We estimated "perception of new daily cases (y)" by assuming y is a function of "actual new daily cases (x)". We denoted: $\frac{dy}{dt}$ = (x-y)/14 as long as x > y; and $\frac{dy}{dt}$ = (y-x)/14 as long as y >= x.
2. We calculated "percentage change of new daily cases (z)" by assuming: z = (x/y) – 1
3. We assumed z is a function of "percentage change in contact frequency given no vaccination (j)" as appears in the graph.

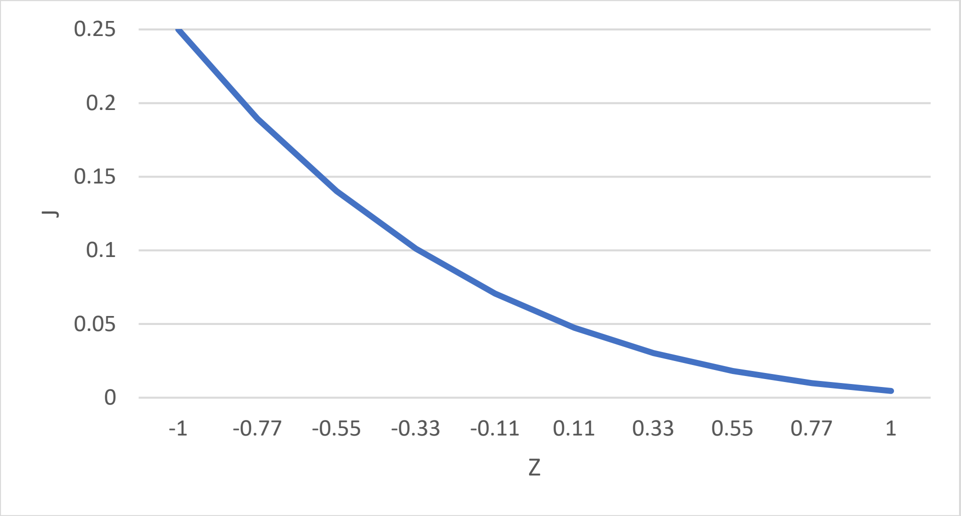

4. We assumed that for the vaccinated people, the "percentage change in contact frequency given vaccination (k)" is double the size of j.
5. We multiplied (1+j) with reproduction number of the non-vaccinated group and (1+k) for the vaccinated group.
